# Characterization of a novel *Xenopus tropicalis* cell line as a model for in vitro studies

**DOI:** 10.1002/dvg.20822

**Published:** 2011-12-27

**Authors:** Ludivine Sinzelle, Raphaël Thuret, Ho-Yon Hwang, Bérénice Herszberg, Elodie Paillard, Odile J Bronchain, Derek L Stemple, Sophie Dhorne-Pollet, Nicolas Pollet

**Affiliations:** 1 Institute of Systems and Synthetic BiologyGenopole, CNRS, Université d'Evry Val d'Essonne, Genavenir 3 - Genopole campus 3, 1, rue Pierre Fontaine, F-91058 Evry, France; 2 Faculty of Life Sciences, University of ManchesterOxford Road, Manchester, United Kingdom; 3 Vertebrate Development and Genetics, Wellcome Trust Sanger InstituteWellcome Trust Genome Campus, Hinxton, Cambridge CB10 1SA, United Kingdom; 4 Neurobiologie and Développement CNRS UPR 3294, Institut de Neurobiologie A. Fessard; Bât. 445, Université Paris Sud91405 ORSAY Cedex, France; 5 Institut National de la Recherche Agronomique (INRA), Unité Mixte de Recherche (UMR) 1313, Génétique animale et Biologie intégrativeF-78352 Jouy-en-Josas, France

**Keywords:** amphibian, transposon, thyroid hormone, frog

## Abstract

Cell lines are useful tools to facilitate in vitro studies of many biological and molecular processes. We describe a new permanent fibroblast-type cell line obtained from disaggregated *Xenopus tropicalis* limb bud. The cell line population doubling time was ∼ 24 h. Its karyotype was genetically stable with a chromosome number of 2*n* = 21 and a chromosome 10 trisomy. These cells could be readily transfected and expressed transgenes faithfully. We obtained stable transformants using transposon-based gene transfer technology. These cells responded to thyroid hormone and thus can provide a complementary research tool to study thyroid hormone signaling events. In conclusion, this cell line baptized “Speedy” should prove useful to couple in vitro and in vivo biological studies in the *X. tropicalis*frog model. genesis 50:316–324, 2012. © 2011 Wiley Periodicals, Inc.

*Xenopus* cell lines can provide many uses to biologists and geneticists (Smith and Tata, 1991). The major advantage of *Xenopus* cell culture is its simplicity compared with the complex cellular populations studied in the context of a whole animal equipped with systemic physiological regulation. Additionally, cell lines are genetically homogenous and offer an unlimited quantity of biological material. This is why several biologists reported the isolation and cultivation of *Xenopus laevis* cells (Anizet *et al.*, 1981; Asashima *et al.*,^1986^; Fukui *et al.*, 1992; Godsell, 1974; Miller and Daniel, 1977; Nakajima *et al.*, 2000; Nishikawa *et al.*, 1990; Pudney *et al.*, 1973; Smith and Tata, 1991). However, there is no characterized *X. tropicalis* cell line described to date.

Here, we present a first description of a *X. tropicalis* cell line. We illustrate the use of these cells for transfection studies, for genome engineering using transposases and as a tool to study the thyroid hormone signaling pathway.

We obtained a primary cell culture named 91.1.F1 from *X. tropicalis* tadpole hindlimbs. These cells displayed an homogenous epithelioid morphology, like the majority of amphibian cell lines described (Anizet *et al.*, 1981). The mean population doubling time (PDT) for 91.1.F1 was around 90 h. At the fourth passage after thawing a frozen cell sample (see materials and methods), we identified two groups of cells from their karyotype (Fig. 1a). The majority of metaphase spreads (63%) was diploid and exhibited the expected chromosome number (2*n* = 20). A second population of cells contained an additional chromosome 10 (trisomy 10). At the ninth passage, we obtained a stable population of these cells, thereafter named “Speedy.”

**Figure 1:**
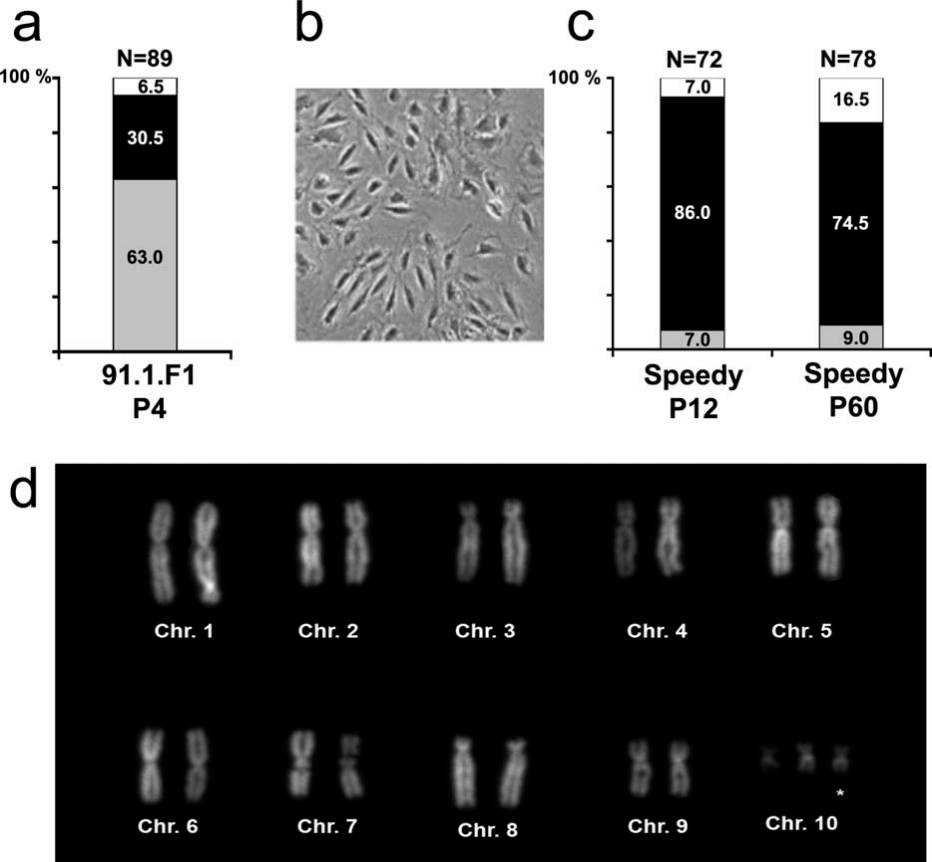
Biological and cytological characterization of the primary cell line 91.1.F1 and the secondary Speedy cell line originating from a *X. tropicalis* limb. (a) Chromosome assay on 91.1.F1 cells at passage 4. Histogramme color code is gray: 20 chromosomes per metaphase; black: 21 chromosomes; white: less than 20 or more than 21 chromosomes. (b) Morphology of Speedy cells in phase-contrast microscopy. (c) Chromosome assay on Speedy cells at passages 12 and 60. Histogram color code as in a. (d) Karyotype of Speedy cells.

Speedy cells were morphologically like fibroblasts with a PDT of ∼ 24 h (Fig. 1b). At passages 12 and 60, the majority of cells exhibited a trisomy of chromosome 10 (2*n* = 21; Fig. 1c). Speedy cells were maintained in vitro over 60 passages (a period of 6 months) without alteration or obvious chromosomal damage during culture (Fig. 1d). Frozen cells were kept over a year and displayed the same karyotype upon revival and after five additional passages.

As Speedy cells contained 21 chromosomes, we checked the identity of these chromosomes by mapping centromeric markers (Khokha *et al.*, 2009). We observed hybridization signals on the centromere of every chromosome pair using each centromeric specific probe (Fig. 2a). Moreover, we confirmed the trisomy 10 (Fig. 2b). Therefore, Speedy cells contain the 10 distinct *X. tropicalis* chromosome pairs.

**Figure 2:**
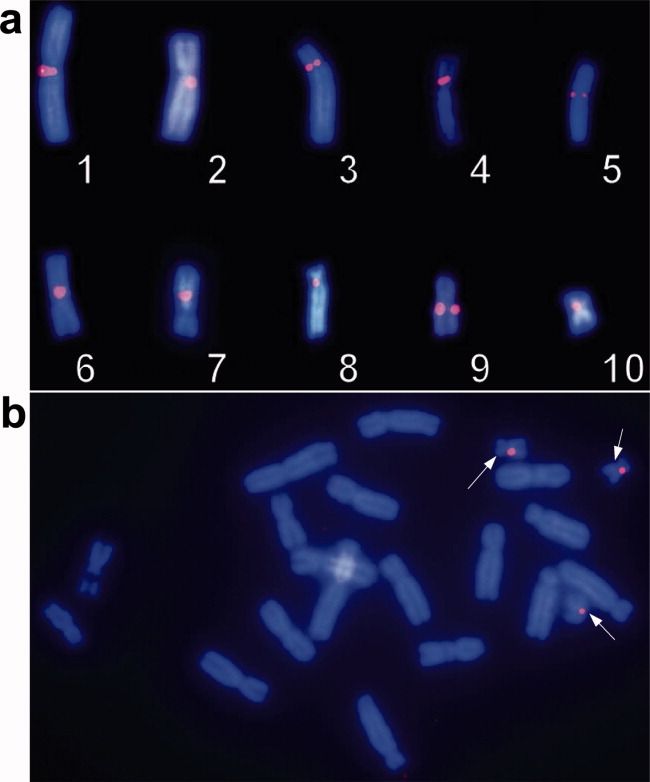
Cytological localization of centromeric markers on *Speedy* chromosomes by FISH. (a) Probe names and corresponding genes (Khokha *et al*.,^2009^: Chr1/LG1 : *mast3*; Chr2/LG6 : *epb41*; Chr3/LG8 : *gemin5*; Chr4/LG7 : *znf423*; Chr5/LG9 : *olig3*; Chr6/LG2 : *fbxl7*; Chr7/LG4 : *mat1a*; Chr8/LG5 : *naif1*; Chr9/LG3 : *stat4*; Chr10/LG10 : *ezh1*. (b) Representative image of FISH analysis of chromosome 10 centromeric probe performed on Speedy cells. The white arrows show fluorescent signals at the centromeric area of all three chromosome 10.

## TRANSFECTABILITY OF SPEEDY CELLS

**Figure 3:**
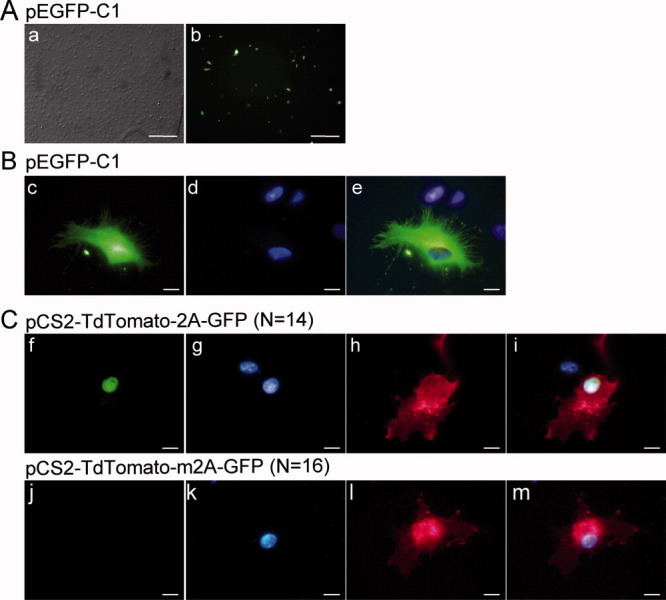
Expression and cellular localization of fluorescent proteins in Speedy cells. (a) Transfectability of speedy cells at 48 h after transfection of pEGFP-C1 plasmid. Scale bar represent 600 μm. (b) Representative images of fluorescent cells transfected with the pEGFP-C1. (c) Representative images of fluorescent cells transfected with the pCS2-TdTomato-2A-GFP and the pCS2-TdTomato-m2A-GFP. (f, j) EGFP fluorescence; (g, k) DAPI staining; (h, l) TdTomato fluorescence; (i, m) Merge of EGFP, DAPI and TdTomato fluorescence. ≪ N ≫ indicates the number of cells observed from two independent transfection experiments. Scale bars represent 10 μm.

We tested the transfection capacity of speedy cells to express exogenous genes using the peGFP-C1 plasmid encoding the green fluorescent protein. We used four commercial lipotransfection reagents, and we analyzed GFP expression using flow cytometry (see materials and methods). Less than 10% of cells expressed significant levels of GFP in all cases. The highest transfection efficiency (6.3%) was obtained using Lipofectamine™, and without fetal bovine serum (Supporting Information Table 1 and Fig. 3a). We observed a homogenous fluorescence throughout both the nucleus and cytoplasm in transfected cells, as expected for the eGFP protein expression (Fig. 3b).

Next, we investigated the ability of Speedy cells to express a protein into a particular cellular compartment. We transfected a biscistronic reporter construct, pCS-TdTomato-2A-GFP, encoding a polyprotein composed of a membrane-localized red fluorescent protein (Myr-TdTomato) and a nuclear-localized green fluorescent protein (eGFP-histone 2B (Trichas *et al.*, 2008). These two coding sequences are separated by a cis-acting hydrolase element (2A viral sequence) enabling cotranslational self-processing. This processing is efficient in human and chicken cells as well as in transgenic mice (Trichas *et al.*, 2008). Speedy cells transfected with the pCS-TdTomato-2A-GFP emit a green fluorescence localized exclusively to the nucleus (Fig. 3c, panel f), whereas the red fluorescence was observed within the cytoplasm, but predominantly in the plasma membrane or in cytoplasmic organelles (Fig. 3c, panel h). These observations are in agreement with previous descriptions in other cell types (Trichas *et al.*, 2008). We could not detect any green fluorescence in Speedy cells transfected with the pCS-TdTomato-m2A-GFP negative control construct (Fig. 3c, panel j), whereas the red fluorescence was distributed in the plasma membrane, the cytoplasm and the nucleus (Fig. 3c, panel l). In conclusion, the 2A peptide sequence was functional in Speedy cells, which were able to correctly address the fluorescent proteins to their respective cellular compartments.

## SPEEDY CELL LINE AS A MODEL FOR SOMATIC TRANSGENESIS USING DNA-BASED TRANSPOSON SYSTEMS

PiggyBac (PB) and Sleeping Beauty (SB) are transposon systems used to modify the genomes of vertebrates (Ivics and Izsvak, 2010). SB is useful to generate germline transmission of transposon transgenes in *Xenopus* (Doherty *et al.*, 2007; Sinzelle *et al.*, 2006; Yergeau *et al.*, 2009). However, transgene insertion is not triggered by the expected “cut and paste” mechanism of SB but by a complex noncanonical integration mechanism. This unusual integration mechanism was only described for the SB transposon system and was not a general property of transposon-mediated transgenesis in *Xenopus*.

**Figure 4:**
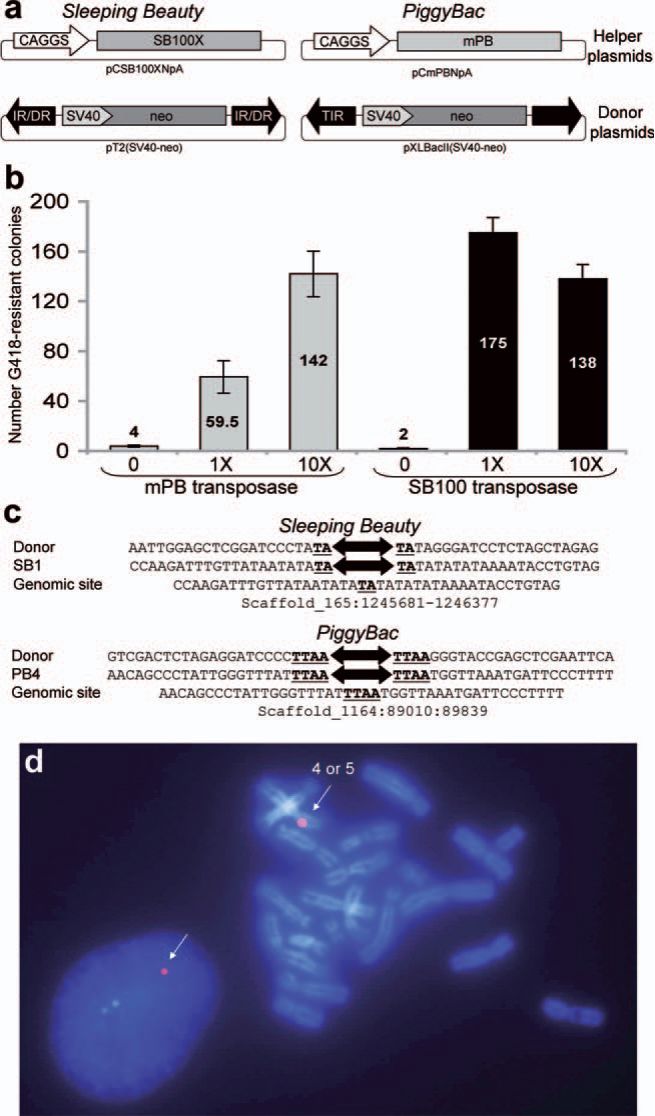
Transpositional activity of SB and PB transposon systems in Speedy cells. (a) Schematic representation of the *mPB* and *SB100* transposon systems. The transposase-encoding plasmids (helper plasmids) expressing SB100 and mPB transposases are pCSB100XNpA and pCmPBNpA, respectively. The donor plasmids for *SB100* and *mPB* transposon systems are pT2(SV40-neo) and pXLBacII(SV40-neo), respectively. (b) Transpositional activities of *SB100* and *mPB* in Speedy cells. The transposition rate was calculated as the ratio between the numbers of resistant colonies obtained in the presence of transposase-expressing construct versus in the absence (*N* = 3 ± SEM). Various amount of helper plasmids, namely 1X and 10X representing 150 ng and 1.5 μg, respectively, were tested: (c) Sequence of integration sites of m*PB* and *SB100* within Speedy gDNA. The TSDs specific to each transposon system (TA for SB100 and TTAA for mPB) are underlined. (d) Representative images of FISH experiments on PBcl6 cell line generated by *mPB* transposition. An interphase nucleus is represented on the left panel whereas a metaphasic spread is shown on the right panel. The white arrows show fluorescent signals.

We tested the transposition activities of SB100 and mPB transposases in Speedy cells to know if they could catalyze bona fide transposition in the *Xenopus* genome. Transposition efficiencies were assessed using a classical colony count assay of G418-resistant clones (Ding *et al.*, 2005; Ivics *et al.*, 1997). We transfected cells with a plasmid encoding a nonautonomous transposon containing a neomycin resistance gene (donor plasmid), along with a transposase-encoding plasmid (helper plasmid; Fig. 4a; Izsvak *et al.*, 2009). We tested two amounts of transposase-expressing constructs for a fixed amount of donor plasmids (Fig. 4b). Using a smaller amount of transposase (1X), we obtained a 14.8-fold increase of neomycin-resistant colonies for PB and a 87.5-fold increase for SB in comparison with the number of colonies obtained with transfection of the donor plasmid alone (Fig. 4b). When we increased the amount of transposase by 10-fold, the transposition efficiency of mPB improved 2.4-fold. However, increasing SB100 transposase expression resulted in reduced transposition efficiency compared with that obtained at the lower amount (1.2-fold decrease). The highest transposition activity was reached with SB100 using 150 ng of donor plasmid for 1.5 μg of helper plasmid.

We recovered the genomic DNA (gDNA) flanking the integrated transposons by inverse PCR to examine the molecular signatures of SB100 and mPB transposition events. Sequence analyses of the inverse PCR amplicons revealed that both mPB and SB100 integrations resulted in the characteristic TTAA-tetranucleotide and TA dinucleotide TSD, respectively (Fig. 4c) (Supporting Information Table 2). Therefore, SB100 and mPB mediate transgene insertion by transposition in Speedy cells, with the expected molecular signature on both integrated transposon junctions.

Finally, to assess the stability of transgene integration and to investigate transposon insertion sites within the Speedy cell genome, a solitary G418-resistant colony resulting from a PB transposition assay was expanded. This transgenic cell line was grown for more than 2 months without morphological change. We verified the chromosomal integration by FISH using a SV40-neomycin cassette probe. A unique hybridization signal was visualized on interphase nuclei (18/25 interphase nuclei), suggesting that a single integration occurred (Fig. 4d). This result was confirmed on metaphasic spreads (9/12 spreads) where a single FISH signal was observed on the short arm of chromosome 4 or 5 (Fig. 4d).

Altogether, we obtained stable transfectant of Speedy cells using transposition-mediated integration and subcloned the transformed cells. Moreover, we provide the first evidence that canonical transposition of SB occurs in *X. tropicalis* and that PB, which was never tested in *Xenopus,* can also be active in *X. tropicalis*.

## SPEEDY CELL LINE AS A MODEL TO STUDY THE THYROID HORMONE SIGNALING PATHWAY

The availability of a homogeneous cell population may help linking in vitro studies at a cellular resolution with in vivo studies in whole organisms. To further illustrate the potential use of the Speedy cell line, we asked if we could use it as a model to investigate the cell autonomous properties of the thyroid hormone signaling pathway.

**Figure 5:**
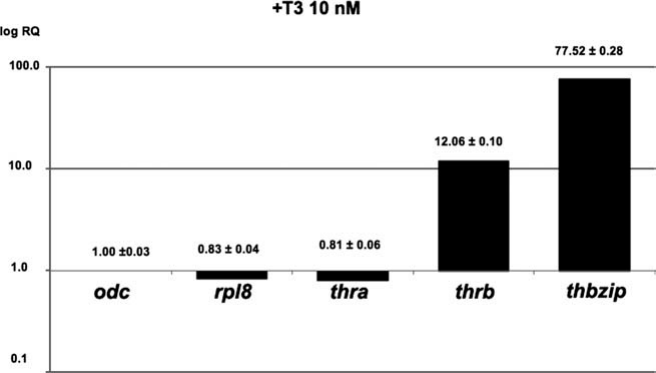
Speedy cells response to TH treatment. The relative quantity of the *rpl8*, *thra*, *thrb*, and *thbzip* mRNA after a 7.5 h of treatment using 10 nM T3 is indicated. The values given are means of three measures. Normalization was performed based on *odc* transcript levels in treated versus untreated cells.

As Speedy cells were established from a hindlimb of a prometamorphic tadpole, we investigated their response to TH treatment. During spontaneous metamorphosis of *Xenopus*, it has been shown that moderate changes occurred in thyroid hormone receptors α (*thra*) gene expression whereas an important upregulation was observed for thyroid hormone receptor β (*thrb*) in hind limbs (Opitz *et al.*, 2005). *thbzip* mRNA level is known to be dramatically upregulated at the climax of metamorphosis. As shown in Figure 5, a 10 nM T3 treatment of Speedy cells for 7.5 h induced a 77.5-fold increase in *thbzip* mRNA level compared to that of untreated cells. At the same time, *thrb* mRNA level was increased 12-fold while *thra* transcript levels are not changed. We conclude that Speedy cells express functional THR and were able to respond to T3 by the upregulation of *thbzip* and *thrb.* Thus, speedy cells response to T3 is similar to what was described in tadpoles during both spontaneous and thyroid hormone-induced metamorphosis and mimicked that of the cell line XL-177 (Kanamori and Brown, 1992; Machuca and Tata, 1992). Further work using this cell line will help us to build dynamic models of TH signaling (Troncale *et al.*, 2007).

The vigorous growth of Speedy cells and the number of passages used to date demonstrated that this cell line could be considered a permanent, genetically stable cell line. We could transfect these cells, and even if the efficiency was low, this proved sufficient to study the subcellular localization of fusion proteins, obtain stable transfectants using transposition-mediated integration and subclone the transformed cells. We hope that the cell line described here will prove useful in a variety of other fields. For example, this cell line could be used for in vivo applications such as nuclear transfer after in vitro manipulation, even though aneuploidy may hinder meiosis and germline transmission.

## METHODS

### Primary Cell Culture

A group of rapidly growing Cam4 F3 *X. tropicalis* tadpoles was grown to stage NF55 (±1) and was anesthetized by <10 min incubation in 0.3% MS-222. The tadpoles were washed extensively with MilliQ water, and their hindlimbs were dissected in PBS. The hindlimbs were incubated in 12.5% sodium hypochlorite for 10 s and were washed twice with Hank's buffered saline solution and once with L15 medium (Invitrogen Gibco). Four hindlimbs were placed in L15, dissociated using an Eppendorf micropestle, and then placed in L15 with 10% USA-certified FBS (Gibco) in a 25 cm^2^ tissue culture flask at 28°C. The growing primary cells were split on day 7 and were subsequently expanded to 10 cm tissue culture dishes on day 9. The cells were allowed to grow until day 37 with an exchange of media on day 20. Penicillin/Streptomycin was not used during this early phase of cell growth and isolation. Isolated clusters of densely packed cells were peeled off using a 200-μl tip and were transferred to 24-well plates and were triturated. After a series of gradual expansions, the cells were maintained by splitting every 4–6 days with change of media every 2 days. One of the isolated cells was designated 59-13-2-1.A3, which was estimated to have doubling time of ∼ 3 days. The 59-13-2-1.A3 cells were passaged eight times after the isolation on day 37 and were frozen. After thawing, the 59-13-2-1.A3 cells were further passaged eight times prior to a transfection attempt. The 59-13-2-1.A3 cells were transfected with a nonlinearized plasmid and were grown in 200 μg/ml of G418 for 10 days with change of media every 2 days and then were grown in 50 μg/ml of G418 for 31 more days with media exchange every 4–5 days. Isolated clusters of densely packed cells were peeled off using a 200-μl tip and were transferred to 24-well plates and were triturated. The cells were gradually expanded to 10 cm tissue culture dishes through three passages and were frozen. One of the isolates was designated 91.1.F1, which like all other isolates tested did not retain the transfected plasmid and showed an epithelial morphology and growth rate largely indistinguishable from the 59-13-2-1.A3 cells. The 91.1.F1 cells were thawed and were passaged 11 times at intervals of 4–6 days before being frozen again. The Speedy cell population is derived from the 91.1.F1 population of cells obtained at this point, and passage numbers in the text and figures are numerated after this point as an origin. Subsequently, the 91.1.F1 cells were maintained for more than 1 year thereafter without any obvious morphological changes (>90 total passages since the isolation of the 91.1.F1 cells and >100 total passages since the isolation of the 59-13-2-1.A3 cells).

### Speedy Cell Culture

The population of 91.1.F1 that led to Speedy cells, and Speedy cells themselves were cultured in 67% (v/v) L15 medium adjusted to amphibian osmolarity by dilution with sterile water, (thereafter referred to as aL15), supplemented with 10% heat inactivated fetal bovine serum (FBS, SIGMA) and a cocktail of penicillin G (50 U/ml) and streptomycin (50 μg/ml) (Invitrogen). Cells were cultivated in a 28°C incubator with constant humidity and in ambient atmospheric conditions. Passages were made twice a week.

To measure growth kinetics, cells were plated on 24-well microplates at a density of 4 × 10^4^ cells/well and cultured for 7 days. Cells were counted every 24 h until the plateau phase. Mean values were used to plot a growth curve and to calculate the PDT.

For thyroid hormone treatment, FBS was depleted of T3 by passing through the AG1-X8 resin (Bio-Rad) as previously described (Kanamori and Brown, 1992) to make tFBS. Cells grown to confluence in 10-cm^2^ culture dishes were transferred in aL15 medium supplemented with 10% tFBS containing 10 nM of thyroid hormone (TH; T3, 3,5,3′-l-triiodothyronine, SIGMA). After 7.5 h of treatment, cells were collected and lysed in 500 μl Trizol (Invitrogen). Untreated cells served as controls.

### Chromosome Analysis and Fluorescence *In Situ* Hybridization Coupled with Tyramide Signal Amplification (FISH-TSA)

Chromosome preparation was carried out as previously described (Krylov *et al.*, 2007). Briefly, colcemid (Invitrogen) was added to the culture medium at a final concentration of 0.6 μg/ml. After 4-5 h incubation, cells were harvested, centrifuged, and treated with a hypotonic solution (40 mM KCl) for 20 min. The cell suspension was fixed in methanol and acetic acid (3:1 v/v) and dropped onto microscopic slides. The slides were then mounted in Mowiol-DAPI (4′,6′-diamino-2-phenylindole, 500 ng/ml). The chromosome numbers per spread were counted for over 70 spreads under an oil immersion objective. We adopted the nomenclature where the largest chromosome is labeled 1, and the smallest 10 (Khokha *et al.*, 2009).

DNA fragments used as probes were synthesized by PCR. The 2110-bp PB(SV40-neo) fragment corresponding to the SV40-neo cassette was PCR-amplified using the pXLBacII(SV40-neo) plasmid as a template. PCR reaction was performed in PCR buffer containing 200 μM of each dNTP, 2 mM of the PB Left and PB right primers (Supporting Information Table 3), 1 mM MgCl_2_ and 1 U of *Taq* DNA polymerase (Fermentas) in a 50 μl reaction volume. The cycling procedure was 94°C for 5 min, then 35 amplification cycles (94°C for 45 s, 56°C for 45 s, 72°C for 2 min), followed by a final extension step at 72°C for 10 min. The resulting amplicon was gel-purified using QIAQuick gel extraction kit (Qiagen). Centromeric markers specific of each chromosome were synthesized using the conditions and the primers pairs previously described from *X. tropicalis* gDNA (Khokha *et al.*, 2009).

For FISH probe synthesis, 500 ng of DNA were labeled using DecaLabel DNA labeling kit (Fermentas) allowing incorporation of Dig-11-dUTP nucleotide (Roche). Labeled DNA probes were purified using a Gel extraction kit (Qiagen). FISH coupled with tyramide amplification (FISH-TSA) was performed as described (Khokha *et al.*, 2009). Briefly, metaphasic chromosomes were prepared as described above and the cell suspension was dropped onto microscopic slides. Then, slides were incubated 5 min with pepsin (50 μg/ml in 0.01 N HCl) at 37°C and fixed with 2% paraformaldehyde for 30 min. Following washes, slides were treated with 1% H_2_O_2_ to get rid of endogenous peroxidase activities. Chromosomes were incubated with the labeled probes overnight at 37°C. Visualization of the hybridized probe was accomplished using an antidigoxigenin-POD, Fab fragments antibody (Roche). Amplification of the FISH signals was carried out with a TSA-tetramethylrhodamine kit (NEN, Life Science, Boston, MA). FISH signals were observed and analyzed under a fluorescence microscope with GFP and DAPI filters (GFP: 450–490 nm excitation filter, 510-nm cut-off filter; DAPI: 365 nm excitation filter, 395 nm cut-off filter), equipped with an AxioCam MRm camera, and the AxioVision software for image analysis (Zeiss, Germany). Labeled chromosomes were identified using their p/q arm ratio and relative size. At least, two slides per probe were analyzed and over 25 interphase nuclei or metaphase spreads were observed.

### Plasmids

Transposons and transposase-expressing constructs used in this study were a kind gift of Dr Z. Ivics and Dr Z. Izsvak (Cui *et al.*, 2002; Grabundzija *et al.*, 2010). *Sleeping Beauty100* transposon system includes the helper and donor plasmids, pCSB100XNpA and pT2(SV40-neo), respectively. *PiggyBac* transposon system includes the helper and donor plasmids pCmPBNpA, and pXLBacII(SV40-neo), respectively. cDNAs of genes located on centromeric loci of the 10 chromosomes and specific primers were kindly provided by Dr Vladimir Krylov (Khokha *et al.*, 2009). pCS2-TdTomato-2A-GFP and pCS2-TdTomato-m2A-GFP expressing constructs were provided by Dr Shankar Srinivas (Trichas *et al.*, 2008).

### Lipofection

Three transfection reagents, including Attractene (Qiagen), Lipofectamine™ LTX and PLUS™ reagent (Invitrogen), and TurboFect (Fermentas) were used to transfect Speedy cells. The cell density and the concentration of plasmid DNAs were fixed, according to each manufacturer's protocol. Transfection experiments were performed with and without 10% heat inactivated FBS (SIGMA). The cultured cells were observed at 48 h after transfection and transfection efficiencies were determined by flow cytometry (FacsCalibur Becton Dickinson) at Genethon imaging-cytometry platform.

### Transposition Assays

Transfections were carried out using Lipofectamine™ LTX and PLUS™ reagent (Invitrogen) according to manufacturer's instructions. A total of 3 μl of PLUS™ reagent as well as 6 μl of Lipofectamine™ LTX transfection reagent were used to transfect 3 μg of DNA. Transposition assays were performed as previously described (Izsvak *et al.*, 2009) using the hyperactive version of SB transposase (*SB100*; Mates *et al.*, 2009) and the mouse codon-optimized version of PB transposase (*mPB*; Cadinanos and Bradley, 2007; Cary *et al.*, 1989; Fraser *et al.*, 1995). Briefly, 1 day prior to transfection, 3 × 10^5^ speedy cells were seeded onto six-well plates to achieve 60–80% confluency. We used two amounts of transposase-expressing constructs, 150 ng (1X) and 1.5 μg (10X) for a fixed amount of donor plasmids (1.5 μg). Each transposition reaction was filled up to 3 μg with pEGFP-C1 plasmid (Clontech). Transfections were performed as described above. Two days after transfection, cells were transferred to individual 100-mm plates in selection medium containing 0.5 mg/ml G-418 for 3 weeks. Resistant solitary colonies were either fixed in fixing solution (5% formaldehyde in PBS) followed by methylene blue staining for colony counting assay, or expanded for insertion site analysis and FISH experiments.

### Quantitative Real-Time RT-PCR Analyses

Total RNA was isolated from cultured cells using Trizol according to the manufacturer's instructions. The isolated RNA was treated with RNase-free DNase (Turbo DNase, Ambion) to ensure complete removal of genomic DNA. After DNAse treatment, RNA samples were purified with the MEGAclear purification kit (Ambion). RNA integrity was evaluated using an RNA 6000 nano kit on an Agilent 2100 Bioanalyzer. One microgram of total RNA was subjected to in vitro reverse transcription using a mixture of polydT and random pentadecamer primers (SuperScriptIII, Invitrogen) (Nolan *et al.*, 2006). Products obtained from reverse transcribed RNAs were monitored using a RNA 6000 pico kit on an Agilent 2100 Bioanalyzer. For the quantitative determination of gene expression, the accumulation of PCR products was measured directly by monitoring fluorescence intensity with a StepOne apparatus (Applied Biosystems). Transcripts from the housekeeping genes ornithine decarboxylase 1 (*odc1*) and ribosomal protein L8 (*rpl8*) were amplified to normalize the cDNA content of each sample (Sindelka *et al.*, 2006). The cycle threshold (Ct) values for target and housekeeping genes were determined, and the mRNA ratio of target gene/*odc1* was calculated by using the comparative Ct method (formula 2^−ΔΔCt^). Data were expressed as mean ± SEM. Nucleotide sequences of the specific primers used are provided in Supporting Information Table 3.

### Integration-site Analysis by Inverse PCR

Genomic DNA (gDNA) from individual G418-resistant clones was extracted by standard methods. One microgram of gDNA was digested with *Spe*I, *Xba*I, and *Nhe*I for SB and *Pac*I and *Pvu*I for PB, respectively. Digestion was followed by ligation with T4 DNA ligase under diluted conditions. Nested PCRs amplifying the left and the right flanks of the transposons were performed by using SBITRL1/SBITRR1 primers, followed by SBITRL2/SBITRR2 primers for SB and PBITRL1/PBITRR1 primers, followed by PBITRL2/PBITRR2 primers for PB (Supporting Information Table 3). PCR reactions were carried out in a final volume of 50 μl with 300 ng of template gDNA, 5 pmol of each primer, 0.2 mM dNTPs, and 1 unit Taq DNA polymerase. The cycling procedure was 95°C for 5 min with a hotstart, then 35 amplification cycles (95°C for 45 s, 56°C for 1 min, 68°C for 2 min), followed by 10 min at 72°C. The amplicons were directly sequenced (GATC, Germany).

## Abbreviations:

IR: inverted repeat
chr.: chromosome
RT: reverse transcriptase
ORF: open reading frame
GFP: green fluorescent protein
